# Epidermolytic Hyperkeratosis - case report*

**DOI:** 10.1590/abd1806-4841.20153966

**Published:** 2015

**Authors:** Marcos Takeyoshi Hayashida, Grasiela Lissa Mitsui, Natalia Ivanoff dos Reis, Giovana Fantinato, Domingos Jordão Neto, Ana Maria da Cunha Mercante

**Affiliations:** 1 Hospital Heliopólis - São Paulo (SP), Brazil

**Keywords:** Acitretin, Hyperkeratosis, epidermolytic, Ichthyosis bullosa of siemens

## Abstract

Epidermolytic hipercetarose is a rare genodermatosis, with a prevalence of 1:100.000 to 1:300.000, with autosomal dominant inheritance. We report the case of a 5 year old girlwho presented an hypertrophic verrucous plaques in the neck, under arm, buttocks, knees, pelvis, legs, dorsum of the right foot and elbows. Histological examination of the skin lesions showed typical changes of epidermolytic hyperkeratosis. Because it is an autosomal dominant disorder with complete penetrance, the individual carrying the mutation will necessarily develop the disease. However, in 50% of cases postzygotic mutation occur. The case report emphasizes early diagnosis and differential diagnoses with ichthyosis and other bullous diseases of childhood, as well as discussing the therapeutic possibilities.

## INTRODUCTION

Epidermolytic hyperkeratosis (EHK), bullous congenital ichthyosiform erythroderma of Brocq, ichthyosis bullosa of Siemens or ichthyosis hystrix Curth-Macklin are synonyms for the same disease.^1^ It is a rare dermatosis, with prevalence of 1:100,000 to 1:300,000, of autosomal dominant inheritance, which affects both sexes equally with high rates of spontaneous mutations occurring in up to 50% of the cases.^1,2^ A case of epidermolytic hyperkeratosis in a female child is described, whose diagnosis was confirmed both clinically and by means of a biopsy.

## CASE REPORT

**Figure 1:**
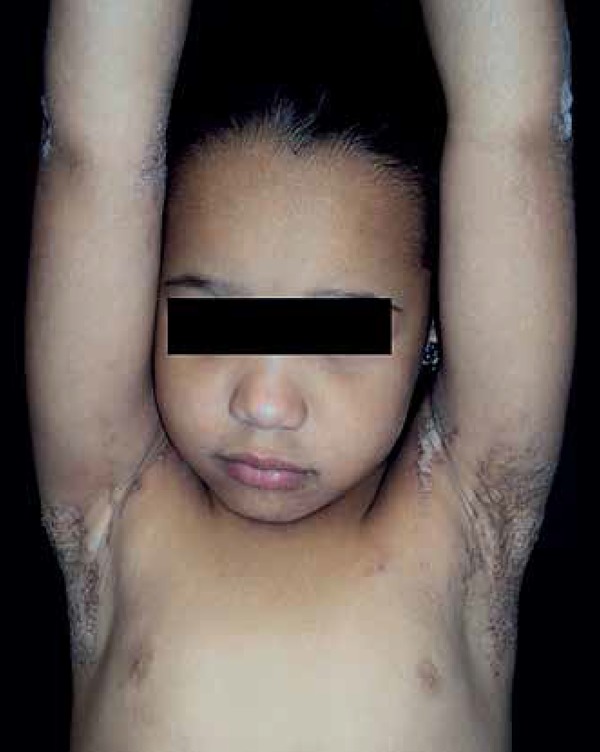
Verrucous and hyperchromic plaques in axillary regions

**Figure 2:**
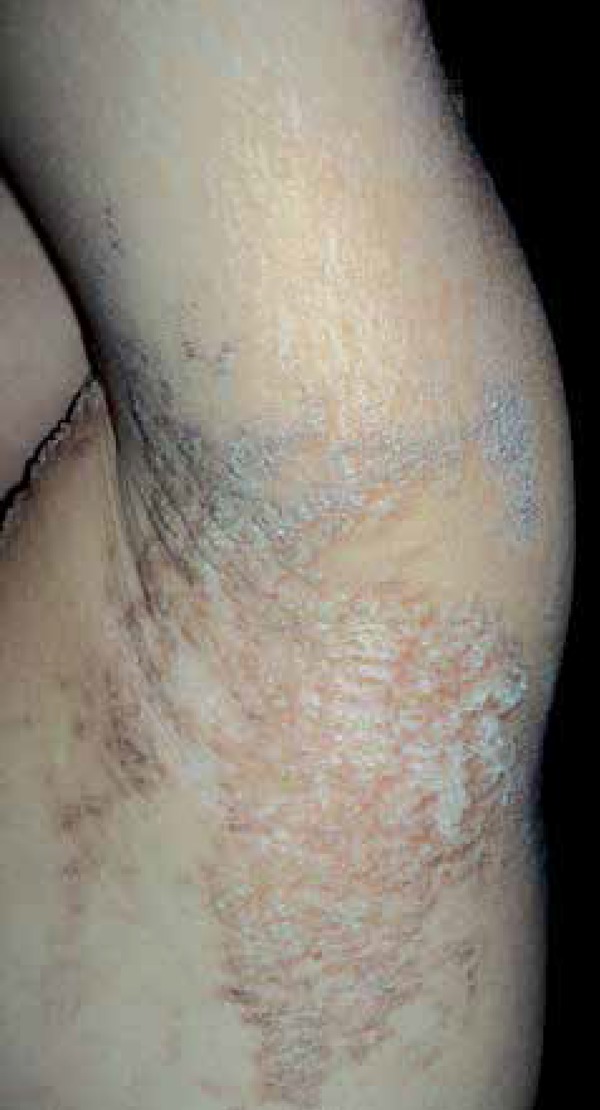
Detail of left axilla affected by verrucous plaques that follow Blaschko lines

**Figure 3:**
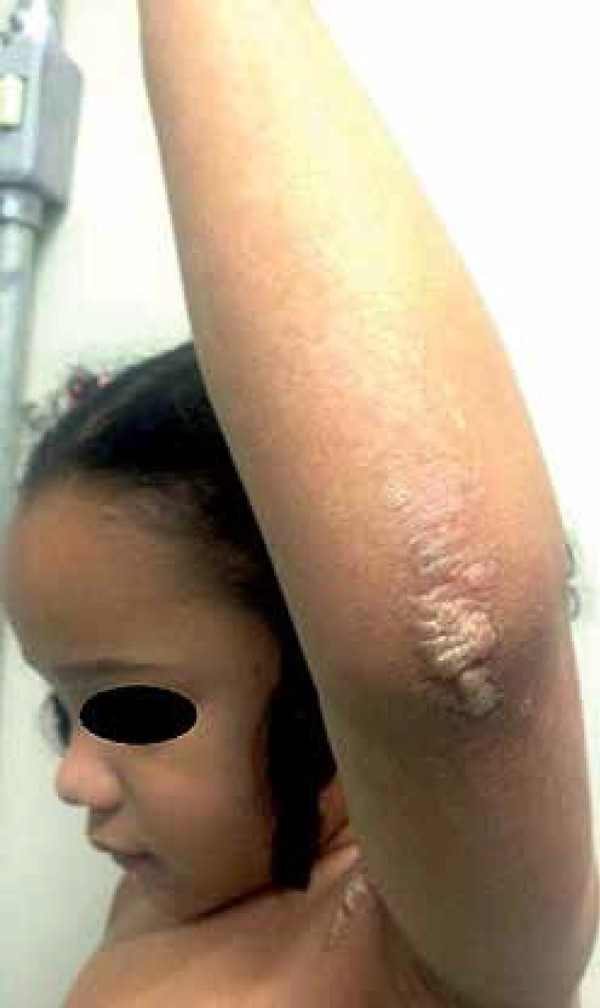
Verrucous plaques grouped on left elbow

**Figure 4:**
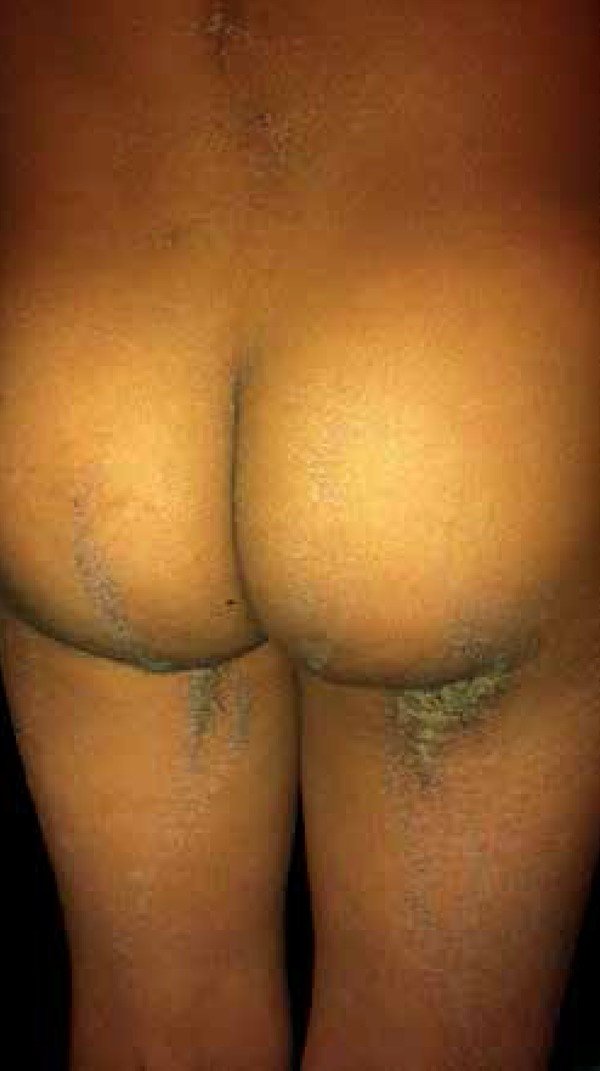
Verrucous and hyperchromic plaques involving buttocks and posterior region of thighs following Blaschko lines

**Figure 5:**
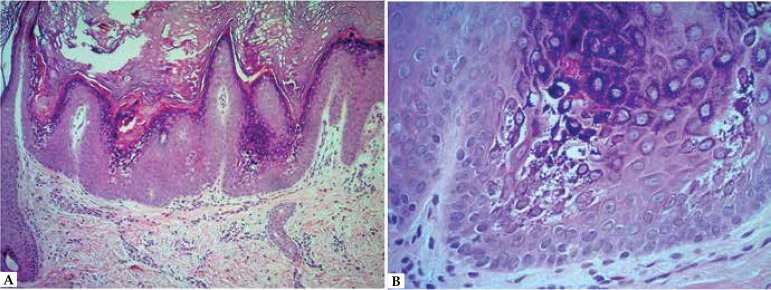
A. HE 100x Epidermis with hyperkeratosis, papillomatosis, hypergranulosis and vacuolated keratinocytes in upper third of epithelium B. HE 400x Keratohyalin granules in vacuolated keratinocytes in upper third of epidermis

Female patient, 5 years and 3 months old, from the state of Bahia. She was born through c-section with no complications. She is the only child of healthy and non-consanguineous parents. Her mother reported that at the time of birth she noticed the presence of blisters and pruritic lesions all over the patient's body and used urea-based topical drugs. At the dermatological examination we observed verrucous and hyperchromic plaques in the cervical region, axillae, buttocks, knees, pelvic waist, legs, dorsum of right foot and elbows (Figures 1 to 4). The histopathological examination demonstrated epidermis with hyperkeratosis, papillomatosis, hypergranulosis, presence of vacuolated keratinocytes, some with keratohyalin granules in the upper third of stratum malpighi, consistent with clinical hypothesis of epidermolytic hyperkeratosis (Figures 5).

## DISCUSSION

EHK has an autosomal dominant inheritance pattern with complete penetrance, although there are some reports of recessive inheritance.^2,3^ The genetic mutation occurs in the gene that codifies cytokeratin 1 (CK1) and/or cytokeratin 10 (CK10), located in chromosomes 17q12-21 and 12q11-13, respectively. These cytokeratins are fundamental components of the cytoskeleton and are expressed in the suprabasal layers of keratinized stratified epithelial tissue. A deficitary cytoskeleton breaks easily, leading to a bullous feature clinically observed in the first months of life. Cytokeratins 1 and 10 have a role in inhibiting cell proliferation, therefore the hyperkeratosis observed would arise from this insufficient inhibition as much as from stimuli of cytokines released during cell rupture.

More severe forms are observed involving the palmoplantar regions in patients with CK1 alterations, in comparison with CK10 mutations. These are represented by milder symptoms, which are usually distributed along Blaschko lines.^2,3^

At birth lesions present with erythrodermia, desquamation and superficial fragile blisters. During the first weeks the skin acquires the typical aspect of the disease, with hyperkeratosis, dark and very thick scales. Palmoplantar keratodermia can be observed, as well as joint contractures secondary to predominance of lesions in flexural areas. Adnexae are generally spared and there is no involvement of internal viscera.^1,2^

There are several forms of clinical presentation. Some classifications were proposed, and since it is a spectral disease, EHK may be classified under the group of ichthyosiform diseases as well as in the group of epidermal nevi. Table 1 divides a sample of patients with EHK into 2 large groups in which the presence or absence of palmoplantar hyperkeratosis is the main distinctive characteristic that distinguishes the groups. The patient of this case report would be classified as NPS2.

**Table 1:** Clinical characteristics of the main subgroups of epidermolytic hyperkeratosis

	NPS - 1	NPS - 2	NPS - 3	PS - 1	PS - 2	PS - 3
	N 11	N7	N5	N25	N3	N1
Plamar-plantar hyperkeratosis	-	-	-	+	+	+
Palmar-plantar surface	Normal	Normal	Superlinear, minimal flaking	Flat	Flat	Cerebriform
Digital contractures	-	-	-	-	+	-
Flaking	Hystrix	Brown	Thin, white	Soft	White, rough	Brownish
Distribution	General	General	General	Local	General	General
Erythroderma	-	-	+	-	+	-
Blisters	+	+	+	Local	+	Neonatal
Abnormal posture (n of affected patients)	1	0	3	0	2	0

**Source:** Ross, et al., 2008^4^

The histology allowed the observation of orthokeratotic hyperkeratosis, papillomatosis, hypergranulosis, acanthosis, vacuolization of granulosa and malpighian cells, and keratohyalin granules dispersed in the vacuolated granular layer.^2,4^

Diagnosis is done clinically, histopathologically and sometimes through molecular biology. In the initial phases a differential diagnosis with bullous epidermolises, staphylococcal scalded skin syndrome and toxic epidermal necrolysis can be done. In hyperkeratotic phases, other ichthyoses are considered while in localized forms, epidermal nevi are considered.

The treatment includes topical keratolytics like urea (10% to 20%), emollients, propylene glycol associated with lactic acid 5%, topical retinoids and liarozole 5% (inhibitor of retinoic acid metabolism); salt immersion baths and hydration may be used.^5^ Another option is the topical use of calcipotriol hydrate + betamethasone dipropionate once a day on lesions, at a maximum of 100g per week.^6,7^ Use of PUVA should be avoided due to increased risk of non-melanoma skin cancer.^8^ In some periods the use of topical antibiotics for *staphylococcus aureus* and antiseptic soaps may be necessary.^2,5^

Acitretin is the main drug used in generalized cases at a dose of 0.5-1mg/Kg/day.^5,8,9,10^ The best response was noticed in patients with CK10 mutation gene, that is, without palmoplantar alterations and with significant improvement in quality of life.^1,2,9^ The mechanism of action of retinoids is still obscure. It seems to generate mutations in the expression of suprabasal keratins involved in EHK.^10^ The use of liarozole cream appears to provide therapeutic benefits and to be safe but its advantage over acitretin is uncertain.^7^

Continuously monitoring of long-term use of oral retinoid is necessary due to skeletal and dyslipidemic abnormalities already reported.^2,9^

Epidermolytic hyperkeratosis is a rare disease that compromises the quality of life of the patient, for the epidermis works as an interface between the individual and the environment. This case report emphasizes early diagnosis, possible differential diagnoses and discusses the most up-to-date therapeutics.
